# Effect of Hydrophilic Polymers on Complexation Efficiency of Cyclodextrins in Enhancing Solubility and Release of Diflunisal

**DOI:** 10.3390/polym12071564

**Published:** 2020-07-15

**Authors:** Mehreen Bashir, Haroon Khalid Syed, Sajid Asghar, Muhammad Irfan, Waleed Hassan Almalki, Salah Ali Menshawi, Ikram Ullah Khan, Pervaiz A. Shah, Ikrima Khalid, Junaid Ahmad, Umar Farooq Gohar, Kok Khiang Peh, Muhammad Shahid Iqbal

**Affiliations:** 1 Department of Pharmaceutics, Faculty of Pharmaceutical Sciences, Government College University Faisalabad, Faisalabad 38000, Pakistan; mehreen.bashir@yahoo.com (M.B.); sajuhappa@gmail.com (S.A.); manipharma1@gmail.com (M.I.); ikramglt@gmail.com (I.U.K.); ikrima_khalid@gmail.com (I.K.); khawajajunaid93@gmail.com (J.A.); 2 Department of Toxicology and Pharmacology, College of Pharmacy, Umm Al Qura University, Makkah 21955, Saudi Arabia; whmalki@uqu.edu.sa; 3 Department of Toxicology in Comprehensive Specialized Clinics Security Forces, Jeddah 21442, Saudi Arabia; captenmd2001@yahoo.com; 4 University College of Pharmacy, University of the Punjab, Lahore 54590, Pakistan; pashah6512@yahoo.com; 5 Institute of Industrial Biotechnology, Government College University, Lahore 54590, Pakistan; dr.mufgohor@gcu.edu.pk; 6 Department of Pharmaceutical Technology, School of Pharmaceutical Sciences, Universiti Sains Malaysia, Minden, Penang 11800, Malaysia; 7 Department of Clinical Pharmacy, College of Pharmacy, Prince Sattam Bin Abdulaziz University, Alkharj 11942, Saudi Arabia; drmmsiqbal@gmail.com

**Keywords:** diflunisal, β-cyclodextrin, hydroxypropyl β-cyclodextrin, hydrophilic polymers, complexation, dissolution rate

## Abstract

The effects of three hydrophilic polymers, namely, carboxymethyl cellulose sodium (CMC-Na), polyvinyl alcohol (PVA) and poloxamer-188 (PXM-188) on the solubility and dissolution of diflunisal (DIF) in complexation with β-cyclodextrin (βCD) or hydroxypropyl β-cyclodextrin (HPβCD), were investigated. The kneading method was used at different drug to cyclodextrin weight ratios. Increases in solubility and drug release were observed with the DIF/βCD and DIF/HPβCD complexes. The addition of hydrophilic polymers at 2.5, 5.0 and 10.0% w/w markedly improved the complexation and solubilizing efficiency of βCD and HPβCD. Fourier-transform infrared (FTIR) showed that DIF was successfully included into the cyclodextrin cavity. Differential scanning calorimetry (DSC) and X-ray diffractometry (XRD) confirmed stronger drug amorphization and entrapment in the molecular cage of cyclodextrins. The addition of PVA, CMC-Na or PXM-188 reduced further the intensity of the DIF endothermic peak. Most of the sharp and intense peaks of DIF disappeared with the addition of hydrophilic polymers. In conclusion, PXM-188 at a weight ratio of 10.0% w/w was the best candidate in enhancing the solubility, stability and release of DIF.

## 1. Introduction

The major challenges of BCS class II (low solubility and high permeability) drugs are poor aqueous solubility and dissolution [1,2,3,4]. Various techniques have been introduced to overcome the limitation of BCS Class II drugs, which include salt formation, lipid-based formulations, particle size reduction, solid dispersions and complexation with cyclodextrins or its derivatives [5,6,7,8,9]. Complexation of poorly soluble drugs with cyclodextrins has become one of the important approaches in formulation development [10,11,12]. Cyclodextrins exhibit basket-like structure with an outer hydrophilic surface, whereas the inner cavity is lipophilic. The whole molecule appears as a cone/torus shape due to limited rotation of bonds connecting the glucopyranose units, providing the opportunity to lodge a variety of suitably sized lipophilic drugs within the cavity [13]. Favorable modifications to the physicochemical properties of poorly soluble drugs, namely, solubility, stability, dissolution rate and bioavailability, could be achieved via complexation with cyclodextrins [14]. Although alpha, beta and gamma cyclodextrins (consisting of six, seven and eight glucopyranose units, respectively) are available, βCD is the most extensively used, which could be attributed to the factors of price, cavity dimensions, approval status and availability [15,16]. Diflunisal is chemically 5-(2′, 4′-difluorophenyl) salicylic acid (C_13_H_8_O_3_F_2_). It is considered a new salicylic acid synthetic analog, in which the difluorophenyl group (at the C5 position of salicylic acid) is responsible for imparting lipophilic character to the molecule [17]. In the literature, the majority of the work was done on the analytical characterization of diflunisal, such as nuclear magnetic resonance studies and ion-selective electrode potentiometry [18,19,20]; however, Zugasti et al. (2009) developed a diflunisal binary system through a coevaporation technique by using a single ratio [21]. Solubility enhancement of DIF via the formation of solid dispersion with polyethylene glycol, inclusion complexes with polyethylene glycol (PEG) and poly e-caprolactone (PCL), solid dispersion with Eudragit RS 100 and RL 100, coprecipitates with PVP K30, βCD inclusion complex, DIF-pyrazinamide and nicotinamide co-crystal were reported [22,23,24,25,26,27]. Enhancement in solubility and dissolution of DIF could ameliorate the bioavailability of DIF.

The addition of hydrophilic polymers was found to improve the solubilization and complexation efficiency of cyclodextrins [28]. In view of the favorable effect as a result of incorporation of hydrophilic polymers, PVA, CMC-Na, and PXM-188, were used for diflunisal in the present study. These hydrophilic polymers could ameliorate the solubilizing and complexation efficiency of cyclodextrins and their derivatives [29,30,31].

PVA maintains a higher concentration of the free drug at the target site, either by augmenting the drug solubility or by shifting the equilibrium between the free drug [D] and [D]–PVA–CD complex towards the former [32]. CMC-Na was reported to enhance the solubilizing efficiency of CDs, thus, improving the complexation efficiency between drug and carrier, as supported by previous data [33]. On the other hand, PXM-188 presents significant improvement in the dissolution profile by impeding the crystalline state of the drug, resulting in an amorphous form of the drug [34]. The purpose of the study was to investigate the effect of carboxymethyl cellulose sodium (CMC-Na), polyvinyl alcohol (PVA) and Poloxamer-188 (PXM-188), on the complexation efficiency of β-cyclodextrin (βCD) and hydroxypropyl β-cyclodextrin (HPβCD), in enhancing the solubility and release of a BCS Class II drug, diflunisal (DIF). The kneading method was used in the preparation of the inclusion complexes. The binary and ternary systems were characterized using scanning electron microscopy (SEM), Fourier-transform Infrared (FTIR), differential scanning calorimeter (DSC) and X-ray powder diffraction (XRD).

## 2. Materials and Methods

### 2.1. Materials

Diflunisal was supplied by AK Scientific (Union city, San Francisco, USA). Beta-cyclodextrin (βCD) and hydroxypropyl β-cyclodextrin (HPβCD) were provided by Roquette (Lestrem, France). Carboxymethyl cellulose sodium (CMC-Na) and Poloxamer-188 (PXM-188) were purchased from Sigma-Aldrich (St. Louis, MO, USA). Polyvinyl alcohol (PVA) was purchased from Daejung (Siheung, Korea). All other materials were of analytical reagent grade and used as received.

### 2.2. Phase Solubility Study

Phase solubility study was conducted according to the method of Higuchi and Connors [35]. Diflunisal in excess amounts was added to 10 mL of aqueous solutions of varying concentrations of βCD or HPβCD with hydrophilic polymers (0, 2.5, 5.0 and 10.0% w/v). The suspensions were placed in a shaking water bath at a temperature of 37 °C for 72 h to reach equilibrium. After centrifuging at 6000 rpm for 30 min, the samples were filtered. The concentration of DIF in the sample was analyzed using a UV spectrophotometer (UV-1700, Shimadzu, Kyoto, Japan) at 251.5 nm after suitable dilution. The stability constant (*Ks*) and complexation efficiency (*C.E*) were calculated using the following equations [36].

(1) $$Ks=\frac{slope}{\mathrm{So}\left(1-slope\right)}$$

where So is the equilibrium solubility of DIF in water; slope is obtained from the plot of DIF concentration against βCD or HPβCD concentrations with or without polymers.

(2) $$C.E=\frac{slope}{1-slope}$$

### 2.3. Preparation of Binary Complexes

The binary inclusion complexes comprising DIF and βCD or HPβCD at 1:1, 1:2 and 1:4 weight ratios were prepared. The drug and cyclodextrins were kneaded in the presence of water and methanol (1:1) for 45 min followed by drying at 45 °C for 24 h. The dried mass was passed through a sieve (number 60) and stored in an air-tight container.

### 2.4. Preparation of Ternary Complexes

PVA, CMC-Na or PXM-188 at concentrations of 2.5, 5.0, 10.0% w/w at dry weight of inclusion complexes were added to binary complexes comprising of DIF and βCD or HPβCD at a 1:2 ratio. The mixtures were kneaded in the presence of water: methanol (1:1) for 45 min, followed by drying at 45 °C for 24 h. The dried mass was then passed through a sieve (number 60) and stored in an air-tight container.

### 2.5. Solubility Studies

The cyclodextrin complexes were added into 20 mL distilled water in glass vials and vortex mixed for 3 min. Then, the vials were placed in a shaking water bath at 30 °C for 72 h. The samples were filtered through a 0.45 µm membrane filter and analyzed using UV-spectrophotometer (UV-1700, Shimadzu, Kyoto, Japan) at 251.5 nm after suitable dilution [37]. Each measurement was repeated in triplicate.

### 2.6. In Vitro Diflunisal Dissolution Studies

The in vitro dissolution studies were conducted using USP dissolution apparatus II (PTWS 3CE, Pharmatest, Hainburg, Germany) paddle method (USP XXXII, 2009). The dissolution medium was comprised of 900 mL of distilled water maintained at 37.0 ± 0.5 °C with a paddle speed of 100 rpm. The quantity of sample equivalent to 10 mg of the drug was weighed accurately and added into each dissolution vessel. Samples of 5 mL were withdrawn at preset time intervals of 2.5, 5.0, 10.0, 15.0, 30.0, 45.0 and 60.0 min, and refilled with fresh dissolution medium. The drug concentration in the sample was analyzed using a UV spectrophotometer (UV-1700, Shimadzu, Kyoto, Japan) at 251.5 nm. Dissolution Efficiency (DE) was determined using trapezoidal method and expressed as percentage of the area of the rectangle divided by the area of 100% dissolution in the same time period. DE% was calculated using the following equation.

(3) $$DE\left(\%\right)=\frac{\int_{0}^{t}yXdt}{y\;100\;Xt}\times 100\%$$

where y is the percentage of dissolved DIF; DE is the area under the dissolution curve between time points *t*_1_ and *t*_2_ expressed as a percentage of the curve at maximum dissolution, *y*_100_, over the same period. It was computed to compare the performance of binary and ternary formulations [38]. The time taken for 50% drug release (*T*_50%_) was obtained from the drug dissolution profiles.

### 2.7. Scanning Electron Microscopy (SEM)

The surface morphology of DIF, βCD, HPβCD, PVA, CMC-Na, PXM-188, binary inclusion complexes and ternary inclusion complexes was observed under scanning electron microscope (JSM-6480, Tokyo, Japan). Electrical conductivity was improved by sputtering a thin gold layer on the samples before imaging.

### 2.8. Fourier-Transform Infrared Spectroscopy (FTIR)

The infrared spectra of samples were obtained using FTIR (BRUKER-Tensor II-ALPHA, Berlin, Germany). The samples (10–50 mg) were directly placed on a sample pan and analyzed over a region from 4000 to 400 cm^−1^ with 10 scans, at a resolution of 1 cm^−1^ and wavenumber accuracy of 0.01 cm^−1^.

### 2.9. Differential Scanning Calorimetry (DSC)

The thermograms of samples were recorded using a differential scanning calorimeter (Universal V4.2E TA Instruments, Newcastle, DE, USA). A sample of 5 mg was crimped in a covered sample pan and heated in an atmosphere of nitrogen (20 mL/min) at a rate of 10 °C/min over the temperature range of 25–400 °C. An empty aluminum pan was used as a reference.

### 2.10. Powder X-ray Diffractometry (XRD)

The diffraction pattern of samples was obtained using an X-ray powder diffractometer (Bruker D-8 Advance, Berlin, Germany). A sample of 10 mg was packed in an aluminum sample container after slight grinding. The required conditions to obtain the diffraction pattern were as follows: Current 30 mA, target Cu, voltage 30 kV, filter Ni, time constant 4 s, scanning 5 to 60 °C, at increments of 0.02 °C.

### 2.11. Statistical Analysis

The results were presented as mean ± standard deviation (SD). The statistical analysis was performed using SPSS^®^ (Version 16, USA). The solubility results were analyzed using one-way analysis of variance (ANOVA). When there was a statistically significant difference, a post hoc Tukey HSD (Honestly significant difference) test was performed. A statistically significant difference was considered at *p* < 0.05.

## 3. Results

### 3.1. Phase Solubility Study

**Figure 1:**
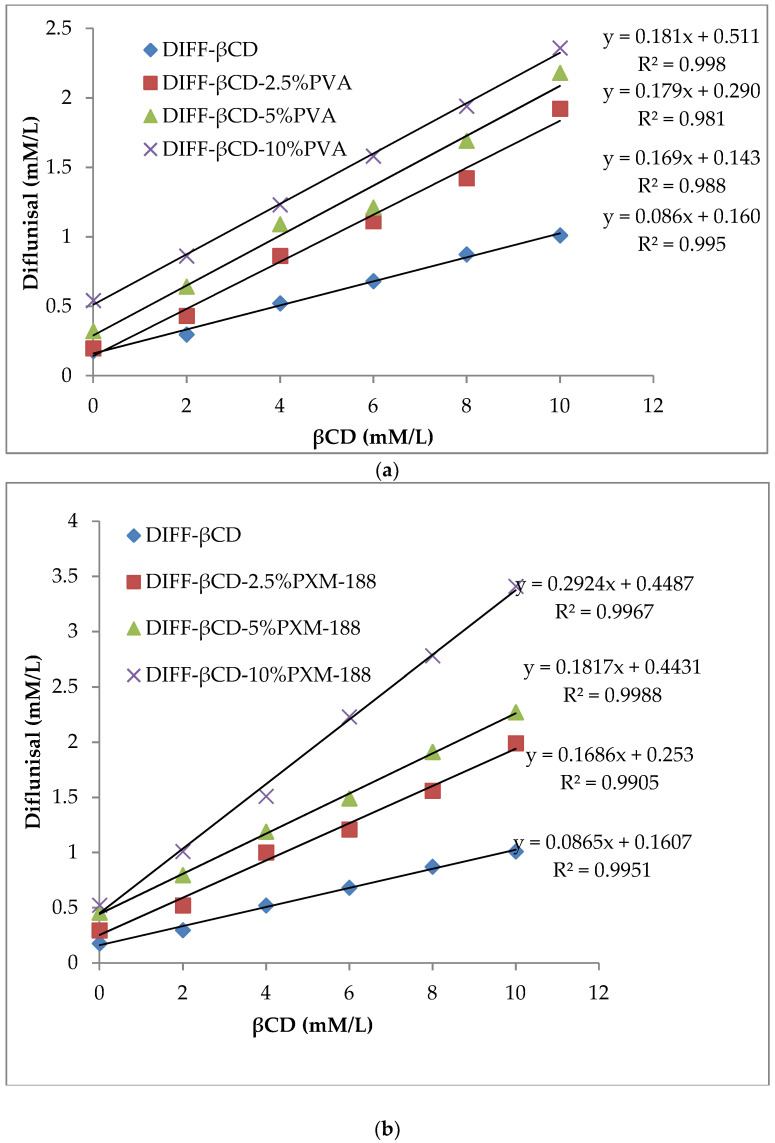

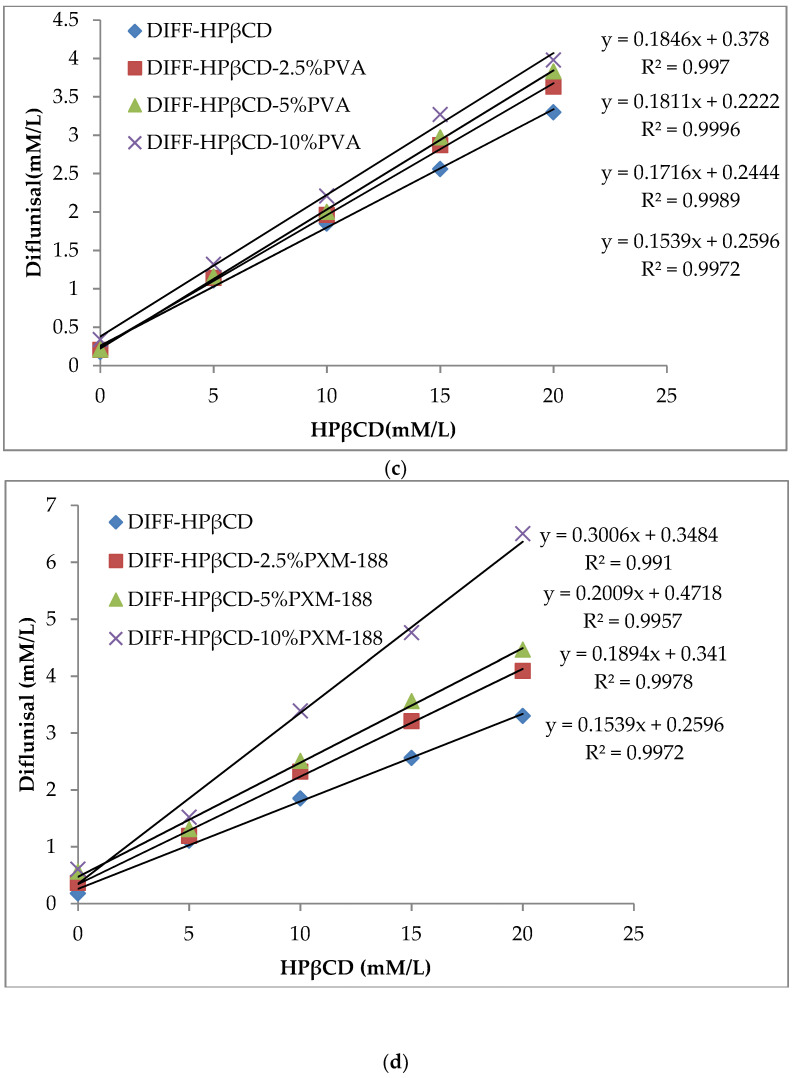
Phase solubility diagram of DIF in aqueous solution of βCD with/without PVA (**a**); with/without PXM-188 (**b**); Phase solubility diagram of DIF in aqueous solution of HPβCD with/without PVA (**c**); with/without PXM-188 (**d**). Mean ± SD, N = 3.

Figure 1a–d demonstrated the proportional increase in solubility of DIF with increases in βCD or HPβCD concentration. A comparatively higher solubility of DIF in HPβCD than βCD could be attributed to a relatively higher solubilization efficiency of HPβCD. The addition of hydrophilic polymers further improved the solubility. PXM-188 yielded the highest value of slope at the same concentration, thus, giving maximum drug solubility. The stability constant and complexation efficiency of both binary and ternary systems of βCD and HPβCD are shown in Table 1. CMC formed gel at this concentration, and hence, was excluded from phase solubility. The ternary system produced more enhanced drug solubilization than the binary system, as indicated by their stability constant values. The reported value of stability constant for improved drug stability ranged from 200 to 5000 M^−1^ [39]. It is favorable to measure complexation efficiency (C.E) as it is a more accurate term in relation to intrinsic solubility determination [40]. 

### 3.2. Solubility Studies

The solubility data of DIF, binary and ternary systems of βCD and HPβCD are given in Table 2. Solubility of DIF was enhanced as a result of complexation with βCD and HPβCD. At a ratio of DIF to cyclodextrin of 1:2, solubility was drastically increased from 44.60 to 450.36 µg/mL for βCD and 907 µg/mL for HPβCD (*p* < 0.05). There was no substantial increase in DIF solubility by increasing the amount of cyclodextrins further (*p* > 0.05). As such, the DIF/cyclodextrin complex at a ratio of 1:2 was chosen for subsequent study. A statistically significant (*p* < 0.05) improvement in DIF solubility was observed by the addition of hydrophilic polymer. The solubility further increased when polymer concentration was raised from 2.5% to 10.0%. The solubility of DIF was the highest with PXM-188, followed by PVA and lastly, CMC-Na. PVA showed better solubility than CMC-Na due to better inclusion efficiency and improved complexation [41]. Maximum solubility was recorded for the DIF/HPβCD/PXM 188 ternary complex (1259.50 µg/mL), which was approximately 28 times that of drug solubility (44.60 µg/mL) and was considered statistically significant (*p* < 0.05). This could be attributed to having the least crystalline features and transformation of its ternary complexes into amorphous form [42].

Valero et al. reported ternary inclusion complexes of naproxen, βCD and PVP, in which the polymer (PVP) has greater affinity for the naproxen/βCD binary systems than the free drug, and surrounded the binary system partially or completely through hydrogen bonding, resulting in pronounced increase in solubility [43]. The presence of CMC-Na did not enhance aqueous solubility of diflunisal considerably in the presence of cyclodextrins. This is further supported by the study of Alexanian et al. (2008) [44]. At low concentrations, CMC-Na interacts with the drug/CD inclusion complex in the same way as with micelles, but upon further increase in concentration, there is no significant increase in solubility, which could be possibly explained on the basis of its higher viscosity.

The ternary systems produced better complexation efficiency and improved solubility than that of binary systems. The polymer interacts with drug/CDs complexes through the outer surface of CD molecules and forms aggregates that are capable of solubilizing drugs and various hydrophobic entities [45].

### 3.3. In Vitro Dissolution Study

**Figure 2:**
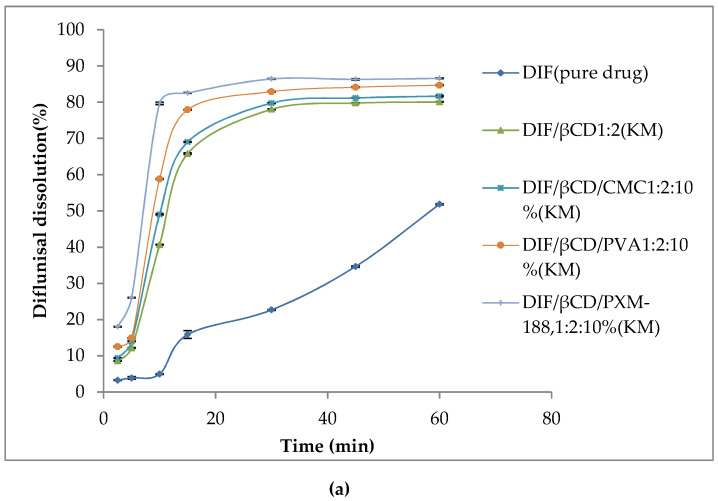

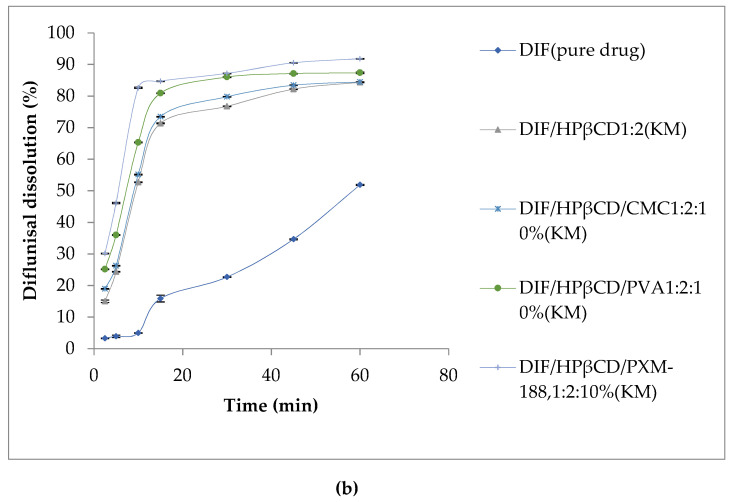
Mean dissolution profiles of DIF, binary and ternary systems with βCD (**a**) and HPβCD (**b**). Mean ± SD, N = 3.

Figure 2a,b showed the dissolution of DIF, binary and ternary complexes with different contents of PVA, CMC-Na or PXM-188. DIF manifested the least dissolution, with approximately 50% of drug released after one hour. It could be observed that the percentage of drug release of both βCD and HPβCD binary systems was comparatively higher than that of DIF. The addition of hydrophilic polymers further increased the drug release rate. The ternary system of DIF/HPβCD/PXM-188 yielded the highest dissolution. The ternary system with CMC-Na and PVA also showed improved dissolution than their corresponding binary systems, but less than PXM-188. The reason behind this is their viscosity, which hinders the diffusion of dissolution medium to the powdered product. Capello et al. (2001) reported that interaction of either the CD molecule or drug–CD complex with the hydrophilic polymers could lead to formation of aggregated hydrates in the solution, increasing the thickness of the diffusion layer and ultimately, slowing the drug release. The dissolution efficiency (DE) and time taken for 50% drug release (*T*_50%_) results are presented in Table 3. The DE and *T*_50%_ values were statistically analyzed using ANOVA (post hoc Tukey HSD test). The rate of in vitro dissolution for DE and T_50%_ values was statistically significant different when DIF and its inclusion complexes were analyzed. A statistically significant difference (*p* < 0.05) was observed when compared to the DE values of the binary kneaded mixture with pure DIF, whereas ternary complexes produced significant differences (*p* < 0.05) and improved DE as compared to the binary kneaded mixture. The longest *T*_50%_ was observed for DIF as compared to its binary and ternary systems. The improved dissolution could be possibly described by the decrease in drug crystallinity of binary and ternary systems.

### 3.4. Scanning Electron Microscopy (SEM)

**Figure 3:**
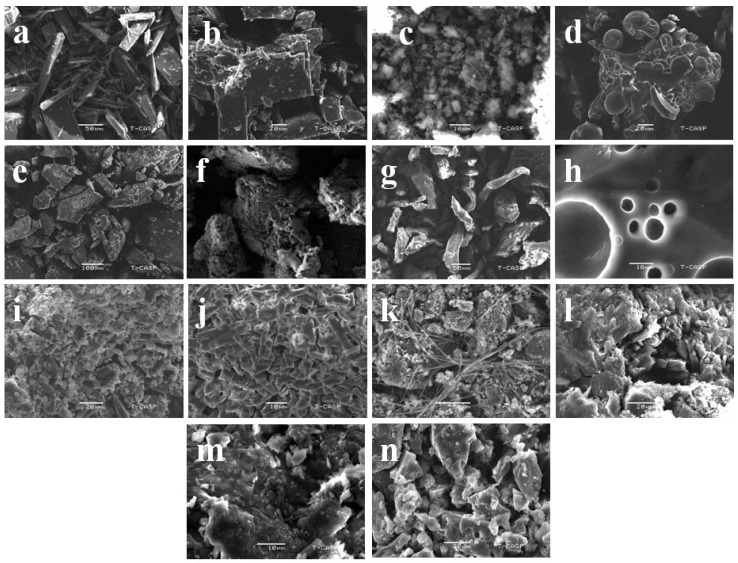
Scanning electron photomicrographs of (**a**) DIF; (**b**) βCD; (**c**) DIF/βCD (1:2); (**d**) HPβCD; (**e**) DIF/HPβCD (1:2); (**f**) PVA; (**g**) CMC-Na; (**h**) PXM-188; (**i**) DIF/βCD (1:2) PVA 10%; (**j**) DIF/βCD (1:2) CMC-Na 10%; (**k**) DIF/βCD (1:2) PXM-188 10%; (**l**) DIF/HPβCD (1:2) PVA 10%; (**m**) DIF/HPβCD (1:2) CMC-Na 10%; (**n**) DIF/HPβCD (1:2) PXM-188 10%.

The scanning electron micrographs of DIF, βCD, HPβCD, PVA, CMC-Na, PXM-188, binary complexes and ternary complexes are given in Figure 3. DIF appeared as crystalline matter with bars of different lengths and widths (Figure 3a). βCD presented as large rhomboidal crystals with non-smooth surfaces (Figure 3b). HPβCD exhibited oval shaped smooth particles of different sizes with concave depression (Figure 3d). The smaller particles were observed to adhere with the larger particles.

The DIF/βCD complex presented a rough surface with indefinite shape (Figure 3c). On the other hand, DIF was found to be included within HPβCD for the DIF/HPβCD complex. The shape of HPβCD changed from globular to a plane lamellar surface due to complex formation. The binary complex appeared as an amorphous structure of miscellaneously dispersed particles with asymmetrical morphology and coarse surface (Figure 3e). The SEM study indicated that the kneading method successfully converted DIF to an amorphous state in CD.

PVA appeared as large smooth surface particles due to its semi crystalline state (Figure 3f). CMC-Na exhibited irregular fiber structure of variable length (Figure 3g). PXM-188 showed a large globular form (Figure 3h). PVA produced a much coarser ternary system with HPβCD than βCD. However, these systems appeared amorphous in the SEM micrographs (Figure 3i and l). In the DIF/βCD/CMC-Na ternary complex, CMC-Na appeared to form a layer over DIF/βCD (Figure 3j), whereas DIF/HPβCD/CMC-Na expressed more amorphous features due to the loss of original shape of the pure drug and HPβCD (Figure 3m). In the DIF/βCD/PXM-188 ternary complex, the crystal structure of PXM-188 disappeared and was adsorbed on the DIF/βCD surface (Figure 3k), retaining little crystallinity. In the case of DIF/HPβCD/PXM-188, the ternary complex appeared as an amalgam and in aggregated form (Figure 3n).

### 3.5. Fourier-Transform Infrared Spectroscopy (FTIR).

**Figure 4:**
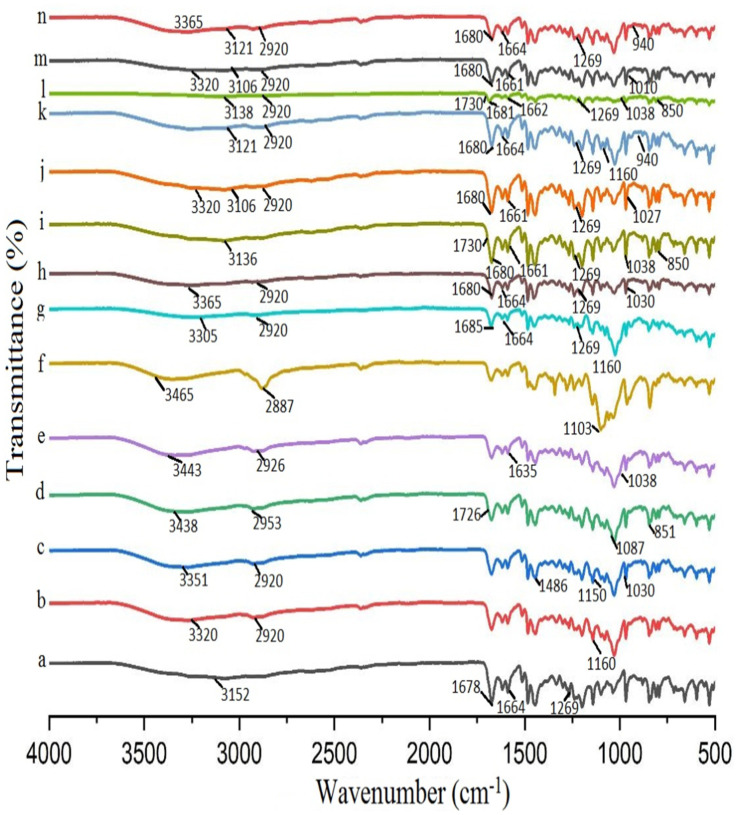
FTIR spectra of (**a**) DIF; (**b**) βCD; (**c**) HPβCD; (**d**) PVA; (**e**) CMC-Na; (**f**) PXM-188; (**g**) DIF/βCD (1:2); (**h**) DIF/HPβCD (1:2); (**i**) DIF/βCD (1:2) PVA 10%; (**j**) DIF/βCD (1:2) CMC-Na 10%; (**k**) DIF/βCD (1:2) PXM-188 10%; (**l**) DIF/HPβCD (1:2) PVA 10%; (**m**) DIF/HPβCD (1:2) CMC-Na 10%; (**n**) DIF/HPβCD (1:2) PXM-188 10%.

FTIR is a practical approach to investigate the drug-polymer interaction and formation of complex Figure 4 showed the FTIR spectra of DIF, CDs, hydrophilic polymers, binary and ternary systems. In the spectrum of DIF, a characteristic broad band appeared at 3152 cm^−1^ (due to aromatic –C–H stretching and OH association and stretching), peaks at 1678 cm^−1^ (due to –C=O stretching), 1664 cm^−1^ (due to stretching in the phenyl nucleus) and 1269 cm^−1^ (due to CF stretch) (Figure 4a). βCD (Figure 4b) and HPβCD (Figure 4c) spectra presented a strong and broad band between 3300–3400 cm^−1^, assigned to stretching vibrations of the hydroxyl group relative to intermolecular hydrogen bonds; the peak at 2920 cm^−1^ corresponded to stretching vibration of the aliphatic C–H group; a band at 1600 cm^−1^ is indicative of deformation vibration of –OH group and C–O stretching vibrations in ester; bands located at 1150 and 1030 cm^−1^ corresponded to the C–O–C stretching vibration [46].

All major peaks related to acetate and hydroxyl groups were observed in the spectrum of PVA (Figure 4d). The band due to intermolecular and intramolecular hydrogen bonding appeared between 3550 and 3200 cm^−1^. The vibrational band appearing between 2840 and 3000 cm^−1^ indicated stretching of C–H from the alkyl group; the peaks appearing at 1750–1735 cm^−1^ represented C=O and C–O stretching from the acetate group; the band at 1087 cm^−1^ was due to –C–C stretching of secondary alcohol; the band appearing at 851 cm^−1^ was due to the rocking mode of the –CH bond of PVA [47]. In the case of PXM-188 (Figure 4f), the spectrum showed peaks at 3465 cm^−1^ (due to -OH stretching), 2887 cm^−1^ (due to –CH stretching) and 1103 cm^−1^ (due to C–O stretching) [29,48]. In the spectrum of CMC-Na (Figure 4e), a strong peak could be visibly seen at 3443 cm^−1^ due to stretching vibration of hydroxyl group and the band area of 1000–1166 cm^−1^ indicated the presence of an ether bond. The carboxylate and methylene moiety gave peaks at 1635 and 2926.8 cm^−1^, respectively [48].

The shifting and intensity changes of bands or peaks gave indication of complexation. There was a significant shifting of DIF peak from 3152 to 3305 cm^−1^ (for βCD) (Figure 4g) and 3365 cm^−1^ for HPβCD (Figure 4h). The shift could be attributed to modification in the hydrogen bonding network. The FTIR spectra of ternary systems signified some characteristic bands of individual elements, thus, reporting the formation of complexes with minor or insignificant alteration of functional groups of individual components. There were no dramatic changes in the peaks pattern of DIF, which confirms the absence of any chemical interaction among the components [49].

In the PVA ternary system (Figure 4i,l), a broad hydroxyl band appeared due to the shifting of the vibrational band (3220–3448 cm^−1^) towards a lower wave number, while the DIF peak at 3152 cm^−1^ was shifted to 3136 cm^−1^ with no significant change at other peaks, but their intensity was reduced, which is indicative of the suitability of ternary systems. The other peaks of PVA at 1726, 1087 and 851 cm^−1^ are shifted to 1730, 1038 and 850 cm^−1^ in the complex, while the peak at 2925 cm^−1^ is found to be absent. Although the main peaks of DIF remained unchanged in the ternary system, the intensity of the peak that occurred due to OH association and stretching and aromatic -CH stretching reduced significantly, which indicates that ternary systems are quite suitable for enhancing complexation efficiency [50]. As for the ternary system with CMC-Na (both with βCD and HPβCD), the characteristic peak of CMC-Na at 1038 cm^−1^ (due to carboxymethyl ether group stretching) diminished considerably and shifted to 1027 cm^−1^ (with βCD) (Figure 4j) and 1010 cm^−1^ (with HPβCD) (Figure 4m), which indicated that CMC-Na might participate in the interaction through this chemical group. The characteristic band of DIF at 3152 cm^−1^, as a result of aromatic –C–H stretching and OH association, was shifted towards a lower wave number at 3106 cm^−1^, while all other peaks did not undergo a significant shift. No new peak was observed in the ternary system, indicative of the compatibility between the drug and carriers.

As for the DIF/βCD/PXM-188 (Figure 4k) ternary system, the characteristic DIF peak at 3152 cm^−1^ shifted to 3122 cm^−1^, an indication of hydrogen bonding resulting in an almost flat region. The DIF peak at 1269 cm^−1^ was significantly reduced and completely masked in DIF/HPβCD/PXM-188, which might be due to insertion of DIF in the molecular cage of cyclodextrin in the presence of PXM-188 (Figure 4n). The peak of PXM-188 at 2887 cm^−1^ vanished, probably due to strong interaction with cyclodextrins. The appearance of a band at 940 cm^−1^ in the spectra of ternary systems of cyclodextrins with PXM-188 was due to the vibration of an α (1→4) glucopyranose ring of cyclodextrins, thus, reinforcing the grafting of cyclodextrin onto the polymeric network. 

The absence of any new peak in the FTIR spectra indicated that there was no chemical incompatibility among the components, only the vanishing and reduction in peak intensity as a result of complexation.

### 3.6. Differential Scanning Calorimetry (DSC)

**Figure 5:**
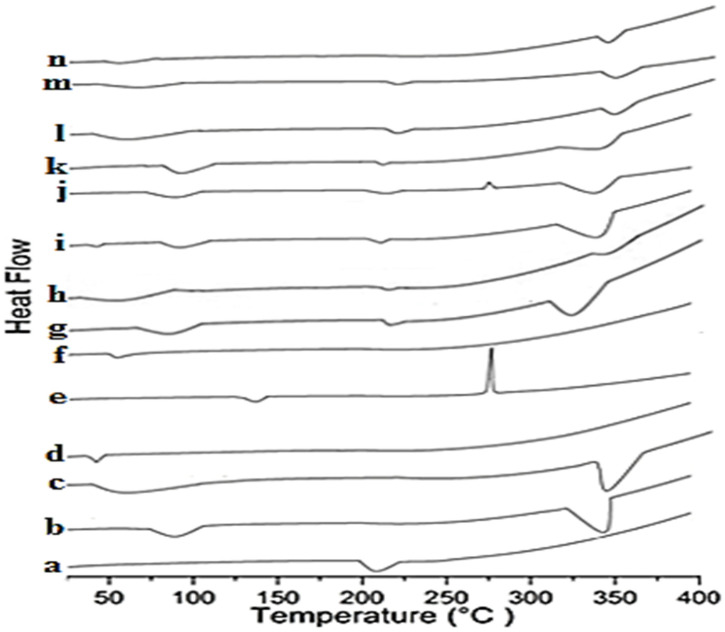
DSC thermograms of (**a**) DIF; (**b**) βCD; (**c**) HPβCD; (**d**) PVA; (**e**) CMC-Na; (**f**) PXM-188; (**g**) DIF/βCD (1:2); (**h**) DIF/HPβCD (1:2); (**i**) DIF/βCD (1:2) PVA 10%; (**j**) DIF/βCD (1:2) CMC-Na 10%; (**k**) DIF/βCD (1:2) PXM-188 10%; (**l**) DIF/HPβCD (1:2) PVA 10%; (**m**) DIF/HPβCD (1:2) CMC-Na 10%; (**n**) DIF/HPβCD (1:2) PXM-188 10%.

The host–guest interaction can be better evaluated by DSC. The melting point peak of the drug is either shifted or disappears as a result of inclusion within the cyclodextrin molecular cage [51,52]. Figure 5 showed the thermograms of DIF, βCD, HPβCD, hydrophilic polymers and the binary and ternary systems. DIF showed a single endothermic peak around 210 °C (Figure 5a). βCD (Figure 5b) and HPβCD (Figure 5c) manifested broad endothermic peaks at 92.4 and 61.8 °C, indicating loss of water molecules due to the dehydration process and melting peaks at 325.2 and 340.2 °C, respectively. PVA showed an endothermic peak at 45.2 °C (Figure 5d). CMC-Na showed an endothermic peak at 130.2 °C and exothermic peak at 274 °C (Figure 5e), while PXM-188 revealed an endothermic peak at about 56.1 °C (Figure 5f).

The reduction in the intensity and shifting of DIF peaks to 212.2 and 214 °C was observed in DIF/βCD and DIF/HPβCD binary complexes (Figure 4g,h). The drug peak was shifted to 213.2 °C for DIF/βCD/PVA (Figure 5i), 214.2 °C for DIF/HPβCD/PVA (Figure 5l), 214.1 °C for DIF/βCD/CMC-Na (Figure 5j) and 216.1 °C for DIF/HPβCD/CMC-Na ternary complexes (Figure 5m). For the DIF/βCD/PXM-188 ternary system, the intensity of the drug peak was remarkably reduced and shifted to 215.1 °C (Figure 5k). An almost flat region was obtained with DIF/HPβCD/PXM-188, indicating reduced crystallinity and improved complexation with HPβCD (Figure 5n).

### 3.7. Powder X-ray Diffractometry (XRD)

**Figure 6:**
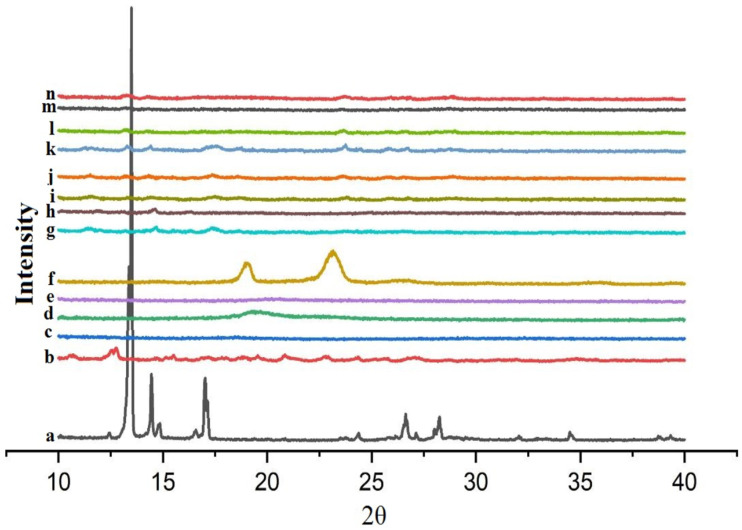
XRD diffractograms of (**a**) DIF; (**b**) βCD; (**c**) HPβCD; (**d**) PVA; (**e**) CMC-Na; (**f**) PXM-188; (**g**) DIF/βCD (1:2); (**h**) DIF/HPβCD (1:2); (**i**) DIF/βCD (1:2) PVA 10%; (**j**) DIF/βCD (1:2) CMC-Na 10%; (**k**) DIF/βCD (1:2) PXM-188 10%; (**l**) DIF/HPβCD (1:2) PVA 10%; (**m**) DIF/HPβCD (1:2) CMC-Na 10%; (**n**) DIF/HPβCD (1:2) PXM-188 10%.

Figure 6 showed the powder XRD patterns for DIF, CDs, hydrophilic polymers and the binary and ternary systems. DIF exhibited crystalline nature, with characteristic peaks at 2θ values of 13.5°, 14.4°, 16.9° and two peaks of lower intensity at 26.5° and 27.9° (Figure 6a). βCD displayed crystalline features (Figure 6b), while HPβCD appeared to be in an amorphous state (Figure 6c). PVA and CMC-Na are amorphous (Figure 5d,e), whereas PXM-188 is crystalline in nature (Figure 6f).

In the binary systems of βCD, the drug peaks at 26.5° and 27.9° vanished and the inclusion complex showed a reduction in the number of crystalline peaks with greater degree of amorphousness (Figure 6g). On the other hand, the drug peaks at 26.5° and 27.9° in the binary system of HPβCD also disappeared. The binary system of HPβCD showed further reduction in the crystalline peaks and expressed greater amorphousness. It is evident from the diffractograms of binary systems that higher C.E could be achieved using HPβCD (Figure 6h).

Diffractograms for ternary systems showed more amorphousness than the binary systems. Although the peaks were detectable in ternary systems, their intensity was reduced and appeared as amorphous. For the ternary system with βCD/PVA, the characteristic peak of PVA at 19.3° broadened, followed by a reduction in intensity, indicating amorphous nature (Figure 6i). For the ternary system with HPβCD/PVA, there was more broadening as compared to that of βCD/PVA and the main peak of PVA at 19.3° completely vanished (Figure 6l). In the βCD/CMC-Na ternary systems, the peak of CMC-Na at 19.8° diminished significantly and an almost flat region was obtained (Figure 6j). In addition, the intensity of drug peak at 13.2° decreased and the inclusion complex appeared to be amorphous. The same trend was observed with HPβCD/CMC-Na; the characteristic peak of CMC-Na at 19.8° vanished and the peak intensity at 13.5° further reduced (Figure 6m). The inclusion complex showed greater amorphousness as compared to the βCD/CMC-Na ternary system. PXM-188 showed two crystalline peaks at 18.9° and 23.1°, while the ternary system of PXM-188/CDs showed a reduction in crystalline peaks of PXM-188 and intensity of drug peak at 13.5° (Figure 6k,n). Some crystalline peaks of PXM-188/βCD vanished and preparations appeared to be amorphous in nature but PXM-188/HPβCD formulations showed greater amorphousness.

The reduction in intensity and broadening of peaks could be better attributed to nanosized particles which cause insufficient diffraction centers, which ultimately lead to peak broadening phenomenon [53]. A correlation between degree of crystallinity and height of peak could be established according to the criterion of Hodge et al. [54]. The release of water molecules (enthalpy-rich) from the cyclodextrin cavity is the motivational force for complex formation [20,55]. These water molecules exhibited higher enthalpy and could not fit properly in the cavity. To stabilize the systems, these more polar molecules were replaced by less polar drug molecules.

The XRD data show that the typical crystalline peaks of DIF were detectable (less in number and reduced intensity). These findings suggest the presence of fewer amounts of crystalline drugs in the system (either binary or ternary, with different extents). DIF appeared to be more amorphous with intense molecular mobility and high energy than the crystalline form, which contributes to improved apparent solubility and dissolution. Binary systems with HPβCD gave better solubility and dissolution data than that of βCD, which is further improved by addition of hydrophilic polymers.

## 4. Conclusions

HPβCD yields better results than those of βCD. Increase in solubility and dissolution of DIF were found with the addition of hydrophilic polymers (PVA, CMC-Na and PXM-188) to binary systems of DIF/βCD and DIF/HPβCD. Ternary systems with PXM-188 presented comparatively more superior performance in enhancing solubility and dissolution of DIF. 

## Figures and Tables

**Table 1:** Stability constant and complexation efficiency results of binary and ternary inclusion complexes.

Inclusion Complexes	Stability Constant (M⁻^1^)	Complexation Efficiency
DIF: βCD	528.6 ± 1.0	0.094
DIF: HPβCD	1014.8 ± 0.1	0.180
DIF: βCD: PVA (2.5%)	1142.5 ± 0.2	0.203
DIF: βCD: PVA (5.0%)	1224.7 ± 1.1	0.218
DIF: βCD: PVA (10.0%)	1241.5 ± 0.5	0.221
DIF: βCD: PXM-188 (2.5%)	1134.3 ± 0.4	0.201
DIF: βCD: PXM-188 (5.0%)	1241.5 ± 2.0	0.218
DIF: βCD: PXM-188 (10.0%)	2317.0 ± 1.0	0.412
DIF: HPβCD: PVA (2.5%)	1150.3 ± 0.6	0.204
DIF: HPβCD: PVA (5.0%)	1241.5 ±1.0	0.221
DIF: HPβCD: PVA (10.0%)	1267.1 ± 1.5	0.225
DIF: HPβCD: PXM-188 (2.5%)	1309.2 ± 0.2	0.233
DIF: HPβCD: PXM-188 (5.0%)	1404.4 ± 0.3	0.250
DIF: HPβCD: PXM-188 (10.0%)	2407.3 ± 0.6	0.428

**Table 2:** Solubility data of DIF, binary and ternary inclusion complexes. Mean ± SD, N = 3.

DIF:CD (w/w)	Solubility (µg/mL)	
	βCD	HPβCD
1:0	44.6 ± 0.02	44.6 ± 0.02
1:1	284.5 ± 0.5	765.5 ± 0.5
1:2	450.3 ± 0.6	907.5 ± 0.5
1:4	500.5 ± 0.5	940.4 ± 0.5
1: 2 (2.5% PVA)	789.6 ± 0.5	965.5 ± 0.5
1: 2 (5.0% PVA)	846.3 ± 0.6	1049.3 ± 0.6
1: 2 (10.0% PVA)	904.1 ± 0.5	1190.3 ± 0.6
1: 2 (2.5% CMC-Na)	692.1 ±0.3	924.3 ± 0.6
1:2 (5.0% CMC-Na)	723.6 ± 0.6	1000.5 ± 0.5
1:2 (10.0% CMC-Na)	800.6 ± 0.5	1089.6 ± 0.6
1:2 (2.5% PXM-188)	791.2 ± 0.7	998.5 ± 0.5
1:2 (5.0% PXM-188)	894.4 ± 0.5	1181.6 ± 0.6
1:2 (10.0% PXM-188)	930.0 ± 0.5	1259.5 ± 0.5

**Table 3:** The dissolution parameters of DIF, binary and ternary systems. Mean ± SD, N = 3.

Samples	DE60 (%)	*T*_50%_ (min)
DIF	24.2 ± 0.3	57.8 ± 0.2
DIF:βCD 1:2	66.8 ± 0.02	11.3 ± 0.03
DIF:HPβCD 1:2	66.7 ± 0.02	9.4 ± 0.01
DIF:βCD 1:2 (10% PVA)	71.6 ± 0.05	8.4 ± 0.01
DIF:βCD 1:2 (10% CMC-Na)	68.5 ± 0.07	10.8 ± 0.01
DIF:βCD 1:2 (10% PXM-188)	76.7 ± 0.07	6.2 ± 0.02
DIF:HPβCD 1:2 (10% PVA)	76.4 ± 0.04	7.65 ± 0.01
DIF:HPβCD 1:2 (10% CMC-Na)	69.3 ± 0.03	9.0 ± 0.01
DIF:HPβCD 1:2 (10% PXM-188)	81.0 ± 0.01	6.53 ± 0.01
